# Work accidents and self-esteem of nursing professional in hospital settings

**DOI:** 10.1590/1518-8345.1632.2872

**Published:** 2017-04-20

**Authors:** Sérgio Valverde Marques dos Santos, Flávia Ribeiro Martins Macedo, Luiz Almeida da Silva, Zelia Marilda Rodrigues Resck, Denismar Alves Nogueira, Fábio de Souza Terra

**Affiliations:** 2 Doctoral student, Escola de Enfermagem de Ribeirão Preto, WHO Collaborating Centre for Nursing Research Development, Universidade de São Paulo, Ribeirão Preto, SP, Brazil.; 3 MSc, Researcher, Universidade José do Josário Vellano, Alfenas, MG, Brazil.; 4 Post-doctoral fellow, Escola de Enfermagem, Universidade Federal de Goiás, Goiânia, GO, Brazil. Adjunct Professor, Escola de Enfermagem, Universidade Federal de Goiás, Jataí, GO, Brazil.; 5 PhD, Full Professor, Escola de Enfermagem, Universidade Federal de Alfenas, Alfenas, MG, Brazil.; 6 PhD, Adjunct Professor, Escola de Enfermagem, Universidade Federal de Alfenas, Alfenas, MG, Brazil

**Keywords:** Occupational Health, Self Esteem, Accidents Occupational, Mental Health, Nursing, Team, Nursing

## Abstract

**Objective:**

to analyze the occurrence of work accidents and the self-esteem of nurses in hospitals of a municipality of Minas Gerais.

**Method:**

descriptive-analytical and cross-sectional study developed with 393 nursing professionals from three hospitals of a municipality in southern Minas Gerais. The Rosenberg Self-Esteem Scale and a questionnaire to characterize the population and work accidents were used for data collection. Data analysis was performed using Person's chi-squared test, Fisher's exact test, Cronbach's alpha, odds ratio and logistic regression.

**Results:**

of the professionals studied, 15% had suffered an accident at work and 70.2% presented high self-esteem. Through the analysis, it was observed that smoking, religious belief and an outstanding event in the career were significantly associated with work accidents. In relation to self-esteem, family income, length of time working in the profession and an outstanding event in the career presented significant associations.

**Conclusion:**

factors such as smoking, religious belief, family income, length of time working in the profession and an outstanding event in the career can cause professionals to have accidents and/or cause changes in self-esteem, which can compromise their physical and mental health and their quality of life and work.

## Introduction

With the changes occurring in the working world in the twenty-first century, professionals now face greater demands in the workplace, increasing their psychological and work burden. With this, the number of accident reported in the workplace has increased. In 2010 there were 709,974 workplace accidents in Brazil, in 2013 this number increased to 717,911 reports^1^ ^-^ ^2^.

Work can often be seen as a causative factor in changes in the conditions of living, illness and death of people. Thus, the work itself, which values ​​and dignifies people, can cause suffering and illness when not performed in adequate conditions that do not favor the psychophysiological capabilities of individuals. This is especially the case for those that work in healthcare, such as nursing professionals^3^.

The representation of nursing professionals in the Brazilian labor market is high. A survey of health professionals conducted in 2015 showed that of the 3.5 million health workers, 1,800,000 were nursing professionals. This highlights the importance of these workers in the health context in the country^4^. 

The work carried out by nursing professionals involves physical proximity of the patient, due to the care process. Thus, these workers are exposed to occupational risk factors, such as physical, biological, chemical, ergonomic and psychosocial factors, which can cause occupational diseases and/or work accidents. Among the most frequent accidents suffered by nursing professionals are accidents with biological materials and with sharps objects^5^.

The exposure of nurses to biological material caused by accidents is a factor that causes suffering at work. These workers, in addition to facing emotional difficulties, such as the fear of falling ill and family and work repercussions, are also subject to embarrassment due to suffering the accident. These factors can lead to personal and social problems, causing changes in well-being and psychological effects, including changes in self-esteem^6^. 

Self-esteem reflects the positive or negative attitudes towards oneself. It is considered a set of feelings and thoughts about self-worth, competence and adequacy^7^. Considering this, it is necessary to relate the occurrence of accidents with nursing professionals to changes in their self-esteem, since emotional problems can affect individuals in their work and in their lives.

In a study conducted in the United States, it was shown that loss of productivity of workers was related to emotional problems, reducing their performance by almost 36%^8^. National and international studies^5^ ^-^ ^8^ have observed the occurrence of accidents at work of nursing professionals and their exposure to psychological harm, such as anxiety, depression, stress and low self-esteem.

Given these findings, due to the problems that work accidents and changes in self-esteem can cause for workers and considering the lack of studies addressing this subject (work accidents and self-esteem), the importance of analyzing the occurrence of workplace accidents suffered by nurses and their self-esteem is justified. This analysis can support knowledge to strengthen the promotion of health of these professionals, leading to better quality of life and mental health, which in turn, can influence the quality of care provided to users.

Thus, the aim of this study was to analyze the occurrence of workplace accidents and the self-esteem of nursing professionals in hospitals of a municipality of Minas Gerais.

## Method

This descriptive, analytical, cross-sectional, quantitative study was developed in three hospitals: a medium-sized private hospital (Institution A, with 289 professionals), a medium-sized philanthropic hospital (Institution B, with 181 professionals) and a small-sized private hospital (Institution C, with 50 professionals), all located in a municipality in the south of Minas Gerais, which provides healthcare to 26 other municipalities of the region. It should be noted that this municipality only possess these three hospitals. The study population consisted of all nursing professionals working in these hospitals, including nursing assistants, nursing technicians and nurses. Thus, the population was 520 workers. 

The following inclusion criteria were adopted: nursing professionals working in the institutions for more than three months, due to the length of experience required. Workers who were on health/maternity leave, or holiday were excluded. Thus, the study sample consisted of 393 nursing staff (Institution A=213, B=143 and C=37). Of the 127 professionals who were excluded from the study, 60 were on vacation, 28 health/maternity leave, 12 had worked for less than three months and 20 refused to participate. It should also be noted that seven workers were excluded due to errors in the completion of the instruments.

Data collection was carried out in the work sector, at times established by the management that did not interfere in the progress of activities, during the period from November 2014 to February 2015. The professionals received sealed envelopes containing two self-report instruments and the consent form, with a brief presentation of the study being made. 

The first instrument was a semi-structured questionnaire, developed by the researchers, which aimed to characterize the population and assess the occurrence of accidents. The variables were selected according to the literature and the aim of the study. This instrument underwent a refining process by five judges with experienced in the study area. Subsequently, a pilot test was conducted with ten nurses in a hospital with similar characteristics to the institutions participating in this study.

The second instrument was the Rosenberg Self-Esteem Scale, developed in 1965 in English, translated and validated for Portuguese in 2001. This instrument uses a Likert type scale with ten questions designed to assess the positive and negative feelings of the individual. The range of the scale score is 10 to 40, with higher scores indicating higher the levels of self-esteem. Therefore, the classification of the self-esteem is measured considering the following cut-off scores: more than 30 points = high self-esteem, 20 to 30 points = normal self-esteem, and lower than 20 points = low self-esteem^9^.

The collected data were entered into a MS-Excel, version 2010, spreadsheet to prepare the database, with double entry subsequently performed to avoid transcription errors. The Statistical Package for the Social Sciences, version 17.0, software was used for the descriptive and inferential statistical analysis. 

Cronbach's alpha coefficient was used to evaluate the reliability of the Rosenberg Self-Esteem Scale. In the univariate analysis, Pearson's chi-squared test and Fisher's exact test were used to verify the existence of associations between the variables: gender, age, marital status, religious belief, number of children, monthly family income, type of housing, consumption of alcohol, smoking, performance of physical activity, chronic illnesses, continuous use of medication, professional category, length of time working in nursing, length of time working in the institution, working hours, working period/shift, having another job, occurrence of an outstanding event in the life and occurrence of an outstanding event in the career, with the variables: occurrence of an accident at work and the measure of self-esteem, and also to check whether the occurrence of an accident at work variable was associated with the measure of self-esteem. 

In this study, a 5% level of significance was adopted, that is, data were statistically significant when *p*<0.05. The odds ratio was estimated and the logistic regression model used for the descriptive variables of the population with the occurrence of an accident at work and the measure of self-esteem. 

Based on Resolution 466 of 2012, this study was approved by the Research Ethics Committee of the Federal University of Alfenas, under Authorization No. 773.900. The hospital institutions authorized the study and the workers that agreed to participate signed the consent form.

## Results

The sample was mainly composed of female professionals (80.4%) aged 30 to 39 years (37.4%, median 35 years), married/with partner (54.7%), with Catholic (78.4%) and family income of R$1,501-3,000 (39.2%, median R$2,500). It should be noted that 75 professionals did not report their monthly family income. It was found that 43.5% of the subjects consumed alcoholic beverages, 11.5% of them were smokers, 38.7% did not practice physical activities, 23.2% had chronic diseases and 32.6% continuously used medication. 

With regard to the professional category, the majority were nursing technicians (75.1%), with length of time in the nursing profession and at the institution of up to 10 years (62.3% and 71.8%, respectively). In addition, the majority worked 42 hours per week (72.5%), during the night period (38.4%) and did not have another job (78.9%). Of the professionals evaluated, 52.9% reported the occurrence of outstanding events in life, the most cited the loss/death of a loved one, the birth of a child/grandchild/relative and the diagnosis of disease in a loved one. A total of 37.4% reported the occurrence of an outstanding event in their career, highlighting the lack of professional recognition, the accumulation of responsibilities/functions and conflicts with the management/coordination.

Of the total of 393 nurses, 60 had suffered some type of accident at work (15%), i.e. 40 accidents in Institution A (10.0%), 18 in Institution B (4.5%) and two in C (0.5%). The accident occurred most frequently during the night shift (35%), followed by the afternoon (33.3%) and morning shift (31.7%). It was observed that 70% of the professionals reported the accident the Communication of Work Accidente (CAT). Of these accidents, 58.3% involved sharp objects, 25.0% contact with bodily fluids, 18.3% falls, 18.3% exposure to radiation/medications, 15.0% contact with furniture/equipment, 10% road accidents and 6.6% burns. Among the causes of these accidents, lack of attention (28.3%), work overload (28.3%), agitation of the patients (26.6%), physical and mental exhaustion (18.3%) and lack of personal protective equipment (13.3%) were reported. It should be noted that more than one response per worker was possible for the last two variables. 

Table 1 presents the only variables that were significantly associated with the occurrence of accidents at work among the nursing professionals. 

**Table 1:** Univariate analysis of the factors associated with the occurrence of accidents at work of hospital nursing professionals, according to the variables: smoking and an outstanding event in the career. Alfenas, MG, Brazil, 2015

Variables	No accident occurrence	Accident occurrence	P-value*	OR**^†^**	95% CI**^‡^**
Smoking					
Yes	33 (73.3%)	12 (26.7%)	0.024	2.273	1.098 - 4.705
No	300 (86.2%)	48 (13.8%)		1.000	
Outstanding event in the career					
Yes	114 (77.6%)	33 (22.4%)	0.002	2.348	1.346 - 4.097
No	219 (89.0%)	27 (11.0%)		1.000

*Pearson's chi-squared test

† Odds ratio

‡ Confidence interval

The univariate analysis of the factors associated with the occurrence of workplace accidents, among all the variables, showed that only smoking and an outstanding event in the career were associated with the occurrence of accidents at work. The participants who smoked were 2.3 times more likely to suffer accidents, and the participants who had undergone some outstanding event in their career were 2.4 times more likely to suffer a workplace accident, according to Table 1. 

Regarding the self-esteem of the professionals, Table 2 shows the distribution of nursing professionals according to the classification observed. 

**Table 2:** Distribution of the hospital nursing professionals according to the classification of self-esteem. Alfenas, MG, Brazil, 2015

Classification of Self-Esteem	f	%
High Self-esteem	276	70.2
Normal Self-esteem	115	29.3
Low Self-esteem	2	0.5
Total	393	100.0

In the assessment of the distribution of the nursing professionals according to the classification of self-esteem, it was found that the majority presented high self-esteem. However, a relevant percentage of professionals were classified as having normal self-esteem and low self-esteem, as shown in Table 2.

In assessing the internal consistency of the Rosenberg Self-Esteem Scale, the internal coefficient of Cronbach's Alpha obtained a value of 0.784. Thus, the internal consistency of the instrument was considered acceptable for the items evaluated and correlated to each other, indicating reliability of the instrument for this study.

In the univariate analysis of the factors associated with self-esteem, only two variables were significantly associated, according to Table 3.

**Table 3:** Univariate analysis of the factors associated with the occurrence of accidents at work of hospital nursing professionals, according to the variables: monthly family income and an outstanding event in the career. Alfenas, MG, Brazil, 2015

Variables	High self-esteem	Low/normal self-esteem	P-value*	OR**^†^**	95% CI**^‡^**
Monthly family income (MFI)					
Up to R$3,000	156 (67.0%)	77 (33.0%)	0.041	1.837	1.021 - 3.306
Above R$3,000	67 (78.8%)	18 (21.2%)		1.000	
Outstanding event in the career					
Yes	91 (61.9%)	56 (38.1%)	0.005	1.866	1.201 - 2.901
No	185 (75.2%)	61 (24.8%)		1.000	

*Pearson's chi-squared test

† Odds ratio

‡ Confidence interval

In this analysis, it was found that the variables monthly family income and an outstanding event in the career were significantly associated with self-esteem. It was evidenced that the nursing professionals who had a monthly family income of up to R$3,000 were nearly 2 times more likely to have low/normal self-esteem, and the professionals who had undergone some outstanding event in their career were nearly 2 times more likely to have low/normal self-esteem, according to Table 3

The logistic regression model of the variables that were significant for the occurrence of occupational accidents and the measure of self-esteem is presented in Table 4. 

**Table 4:** Evaluation of the parameters of the logistic regression model of the independent variables with accidents at work and self-esteem of the hospital nursing professionals. Alfenas, MG, Brazil, 2015

Variables	Parameter	Standard-error	OR*	P-value
Work accident				
Religious belief	1.049	0.499	2.854	0.036
Outstanding event in the career	0.875	0.320	2.399	0.006
Self esteem				
Length of time in nursing profession	0.771	0.287	2.162	0.007
Outstanding event in the career	0.650	0.261	1.916	0.013

*Odds ratio

When analyzing the parameters for all the independent variables, with the occurrence of work accidents through the logistic regression model, it was found that only the religious belief and an outstanding event in the career variables showed statistical significance, p=0.036 and p=0.006 respectively, resulting in a final adjusted model, as presented in Table 4. Thus, the final model showed that the professional who was Catholic was 2.8 times more likely to suffer an accident at work. In addition, the nursing professionals who experienced an outstanding event in the career were 2.4 times more likely to suffer a workplace accident.

 After analyzing the parameters of all the variables with the measure of self-esteem through the logistic regression model, it was found that only the length of time in the nursing profession and an outstanding event in the career variables presented statistical significance, resulting in the final adjusted model, as shown in Table 4. Thus, professionals with over 10 years of professional experience in the field of nursing were 2 times as likely to have low/normal self-esteem and those who experienced an outstanding event in their career were nearly 2 times more likely to have low/normal self-esteem. 

When evaluating the association of the self-esteem variable with the occurrence of work accidents among the nursing professionals, it was clear that there was no significant association between these two variables (p=0.966). 

## Discussion 

It was observed in this investigation that some of the nursing professionals suffered some kind of accident at work. The majority of these accidents were with sharp objects, occurred during the night shift and were reported in the CAT by the professionals. A study conducted in a city of the state of Paraiba, with 39 nursing professionals, showed that 49% of them suffered an accident at work, 84% of the accidents were caused by sharps objects and 79% of the cases were not reported^10^. Another study developed in northeastern Brazil, with 45 nursing professionals, found that 60% of them had accidents and 24% of these occurred during the night shift^11^. Work accidents are often justified by the fact that some institutions do not have effective policies to promote safety at work, leaving workers exposed to risks. The main factors that favor the occurrence of these accidents with sharps objects are linked to the working conditions, highlighting unsanitary conditions and the dangers of handling work material^1^ ^,^ ^12^. Regarding the work shift, professionals who work at night, due to the workload and long working hours, have more risk of developing occupational diseases and being involved in accidents, due to fatigue, physical and mental wear and biological changes in the body^13^. 

After having an accident professionals should record the occurrence. However, it was noted that the number of CAT registrations is still low considering the real health situation of the professionals. This is because some companies neglect aspects related to the health of the workers and their working conditions^1^. 

In this study, the main cause of accidents at work was a lack of attention and work overload. Thus, it appears that diverse factors of the work environment of the nursing professionals may have contributed to the occurrence of accidents. Among these factors, performing tasks quickly, lack of attention during the execution of a procedure, physical and mental fatigue of the worker and work overload have been reported^14^. 

In this study, it was noted that the professional who used tobacco had a higher chance of suffering work accidents. This factor may be justified because the use of tobacco can cause psychiatric disorders such as anxiety and attention deficit, among others^15^. 

It was also found that the professional who was Catholic was more likely to suffer work accidents. This finding may be related to the fact that 78.4% of the professionals in this study were Catholic and, of the 60 workers that had accidents, 52 reported having this religious belief. The result may have been influenced by this factor, however, could also be justified by the nursing care be guided by Christian sentiments, performed with solidarity, compassion and proximity, exposing the professional to psychic burdens and accidents at work^6^.

Another variable that was significantly associated with accidents at work was the occurrence of an outstanding event in the career, with the professionals that had undergone this type of event being more likely to suffer workplace accidents. It should, therefore, be noted that experiencing an event in life or at work can trigger problems in workers, such as emotional stress and mental imbalance, which exposes them to psychic burdens. With this, job insecurity can be presented, making the people more vulnerable to occupational hazards and accidents^16^.

The psychic burdens that favor fatigue of the worker may come from various factors, such as strict supervision, fast rhythm, monotony, repetitiveness of tasks, difficulties in communication, psychological aggression, tension and dissatisfaction. These conditions may worsen the health of workers and generate work-related accidents^17^. 

Regarding the measure of self-esteem of the nursing professionals, it was observed that the majority of them had high self-esteem, which reflects appropriate psychological conditions for work. It can be inferred that there was a percentage of professionals who presented low self-esteem, which can severely limit individual inspiration and achievements. Its consequences may appear indirectly, whereby the professional works without proper care, prone to errors and unhappiness in the work and the personal life^18^.

This study found that family income was significantly associated with self-esteem. Thus, nursing professionals who had a family income of up to R$3,000 were more likely to have low or normal self-esteem. Insufficient remuneration can generate professional insecurity and fear of losing the job, with this contributing to a precarious work regime. This factor may also lead the professional to the need to seek additional employment. Thus, workers sacrifices their leisure and rest time to perform another job^6^ ^,^ ^19^. The importance of the hospitals providing decent wages for the professionals can be perceived, since this can promote better health and work conditions and thus increase the self-esteem and self-confidence of nursing professionals.

The length of time in the nursing profession variable was also significantly associated with self-esteem. It was shown that the professionals who had more than 10 year in their nursing career, were more likely to have low or normal self-esteem. It should be noted that there are aspects in nursing work that can worsen over time. These aspects relate to the everyday life of nursing work, such as stress, requirements and work overload, among others. Over the years engaged in the profession, these factors can cause mental disorders in professional, such as boredom and the feeling of fatigue, resulting in low self-esteem^19^ ^-^ ^20^. 

It was observed that an outstanding event in the career variable was significantly associated with self-esteem. Professionals who had experienced some outstanding event in the career were more likely to have low or normal self-esteem. Many factors that may be associated with these events can cause emotional distress and lead to feelings of helplessness and discouragement, resulting in psychological overload and impaired self-esteem^16^. 

It should be noted that the innumerable loads generated by the occurrence of outstanding events are related to the rapid and repetitive work, lack of interaction of the team, pressure from the management and colleagues and physical and mental fatigue, among others. These situations can place professionals in situations that are not conducive for mental health in the nursing practice^6^. 

When checking for a possible association between the self-esteem and the accident at work variables, it was found that there was no significant association. However, it should be noted that the occurrence of workplace accidents can be a psychological distress factor for professionals. 

Psychological disorders are part of the reality of the health of the worker, influencing the work performance and productivity of professionals, favoring illness and the occurrence of accidents. Therefore, it is important to mention that the complexity of nursing tasks, plus the responsibilities and technical-scientific concerns may contribute to possible changes in self-esteem. Thus, these workers may not be able to perform their tasks safely, impairing their professional performance and exposing them to the risk of accidents at work^1^ ^,^ ^21^.

## Conclusion

It was concluded that some of the professionals evaluated suffered workplace accidents and the majority of them had high self-esteem. Of the variables, smoking, religious belief and an outstanding event in the career showed significant associations with accidents at work and the variables monthly family income, length of time in the nursing profession and an outstanding event in the career were associated with self-esteem. 

This demonstrates the need to promote better working conditions in the hospital environment, since nurses are exposed to occupational risk factors, which can compromise their physical and mental health, leaving them vulnerable to accidents at work and to changes in self-esteem. In this context, it is suggested that measures be taken to promote quality of life and work, through continuing education, psychological support and valorization of the professional. To further investigate the health of nursing professionals, the performance of longitudinal studies on this topic is suggested, aiming to verify the cause-nexus and the cause-effect between work accidents and self-esteem. 

This study presented some limitations, such as the cross-sectional design of the study, which did not allow the cause-effect relationship of the results to be verified; the sampling, due to the collection not having been performed with the total population of hospital nursing professionals; the limited access to some sectors of the institutions, due to their complexity; and the self reported nature of the instruments, as some of the professionals failed to respond to some questions. Considering the above, it can be stated that by adopting the measures suggested, professionals would have adequate working conditions and a healthier life, which would favor the quality of care provided to the health service users.
